# The Main Therapeutic Applications of Cannabidiol (CBD) and Its Potential Effects on Aging with Respect to Alzheimer’s Disease

**DOI:** 10.3390/biom13101446

**Published:** 2023-09-26

**Authors:** Václav Trojan, Leoš Landa, Alexandra Šulcová, Jiří Slíva, Radovan Hřib

**Affiliations:** 1 International Clinical Research Centre, Cannabis Facility, St. Anne’s University Hospital, Pekařská 53, 602 00 Brno, Czech Republic; 2 Department of Pharmacology, Faculty of Medicine, Masaryk University, Kamenice 5, 625 00 Brno, Czech Republic; 3 Department of Pharmacology, Third Faculty of Medicine, Charles University, Ruská 87, 100 00 Prague, Czech Republic; 4 Centre for Pain Management, Department of Anesthesiology and Intensive Care, St. Anne’s University Hospital, Pekařská 53, 602 00 Brno, Czech Republic

**Keywords:** cannabidiol, aging, Alzheimer’s disease, cannabinoids

## Abstract

The use of cannabinoids (substances contained specifically in hemp plants) for therapeutic purposes has received increased attention in recent years. Presently, attention is paid to two main cannabinoids: delta-9-tetrahydrocannabinol (THC) and cannabidiol (CBD). With respect to the psychotropic effects and dependence potential of THC (though it is very mild), its use is associated with certain restrictions, and thus the therapeutic properties of CBD are frequently emphasized because there are no limitations associated with the risk of dependence. Therefore, this review covers the main pharmacodynamic and pharmacokinetic features of CBD (including characteristics of endocannabinoidome) with respect to its possible beneficial effects on selected diseases in clinical practice. A substantial part of the text deals with the main effects of CBD on aging, including Alzheimer’s disease and related underlying mechanisms.

## 1. Introduction

The United Nations General Assembly declared the years 2021–2030 as the “Decade of Healthy Ageing”: a challenge for international cooperation expected at the widest possible levels in both social and medical care and research to improve the lives of older people.

This challenge means paying great attention to the study of all the processes that can accelerate human aging, which is associated with the gradual degeneration of structures and the functioning of vital organs [1], but also, of course, how to deal with the unpleasant symptoms that appear. Attention needs to be focused both on general social and psychological approaches and on finding and validating appropriate (or even innovative) pharmacological treatments.

A growing number of scientific reports indicate that a healthy human microbiome is supported by the so-called “endocannabinoidome” [2]. The name endocannabinoidome has been proposed for the complete set of lipid mediators (the endogenous cannabinoids or endocannabinoids such as anandamide and 2-arachidonoylglycerol) relevant for biochemical and pharmacological signaling through binding to a widespread network of cannabinoid receptors (CB1 and CB2) throughout the body of vertebrates, including man [3,4].

The effects of cannabinoids are highly ubiquitous in the bodies of vertebrates, including humans, because their receptors have been confirmed in the nervous system (cortex, basal ganglia, hippocampus, hypothalamus, cerebellum, spinal cord, spinal cord ganglia), but also in the enteric nervous system, adipose cells, vascular endothelial cells, liver, and gastrointestinal tract. As has been proven many times, the body’s lipoid substances, called endocannabinoids, are used to regulate physiological functions. It is not surprising that attention is paid to investigate not only their physiology but also their pathophysiology, e.g., in pain, inflammation, immune disorders, obesity and other metabolic disorders, cardiovascular and gastrointestinal diseases, and neurodegenerative changes, i.e., conditions occurring frequently in the elderly. To transfer knowledge about the physiology and pathophysiology of the endocannabinoidome (of vertebrates, including humans) into possible therapeutic use, intensive preclinical and clinical research studies are underway (see below). Experimental preclinical research, together with clinical trials (i.e., translational research), is focused in geriatrics on the validation of therapeutic approaches using either certain cannabinoids [5,6,7,8,9] or agents enhancing endocannabinoid activities without binding to cannabinoid receptors (e.g., endocannabinoid biodegradation inhibitors) [10,11].

One of the possible and promising new approaches to aging management involves a substance naturally occurring in the hemp plant called cannabidiol (CBD). In general, the use of hemp-specific substances and their possible therapeutic use have received increased attention during the past few years for the treatment of many diseases or pathological conditions, especially chronic persistent pain [12,13]. CBD is one of the best-studied substances found in hemp, and since the possible advantageous effect of this substance has also been described on aging, we would like to focus on the possible role of CBD in this process.

## 2. Cannabinoids and the Endocannabinoidome

In general, cannabinoids are chemical substances acting primarily on specific cannabinoid receptors, and they are presently classified into three groups: endogenous cannabinoids (endocannabinoids) found naturally in various tissues of vertebrates; herbal cannabinoids (phytocannabinoids) occurring specifically in hemp; and finally synthetic cannabinoids produced chemically [14]. Besides these “classical” types of cannabinoids, presently we also define substances called endocannabinoid-like molecules (ELMS; e.g., palmitoylethanolamide and oleoylethanolamide), which do not activate CB1 and CB2 receptors, and instead of this mechanism, they activate various G-protein-coupled receptors (GPRs) or ion channels, perhaps even nuclear receptors, or affect the effects of cannabinoids by other means [15].

The endocannabinoidome exhibits functions of anti-inflammatory and anti-excitotoxic barriers [6] and can be positively modulated by the administration of either phytocannabinoids or other cannabinoid receptor agonistic ligands [7,8,10,16,17,18,19,20,21,22,23,24] or even by interactions with enhancers of the endocannabinoid activities without binding to cannabinoid receptors, e.g., endocannabinoid biodegradation inhibitors [11,25,26,27,28].

Based on all relevant research data available, U.S. Patent No. 6,630,507 2013 was granted to the Department of Health and Human Services for the medical use of CBD. After five years of experience using cannabinergic therapies, 16 selected experts of the National Academies of Sciences, Engineering, and Medicine evaluated the medical assessments with the result that there is: “I. sufficient scientific evidence for the therapeutic efficacy of cannabinoids for the treatment of (a) pain in adults; (b) nausea and vomiting during chemotherapy; (c) spasticity in multiple sclerosis; II. the average amount of expert evidence for the treatment of (a) secondary sleep disorders; (b) loss of appetite; (c) irritable bowel syndrome; (d) Tourette syndrome; (e) anxiety; (f) post-traumatic stress disorder; (g) cancer; (h) epilepsy; (i) various neurodegenerative disorders. At the same time, the development of new cannabinoid drugs was approved with the following indications: (a) neurological damage in cases of stroke or injury; (b) neurodegenerative diseases such as Alzheimer’s and Parkinson’s disease and HIV infection”. The summary from 10,000 abstracts of the US National Academies of Sciences, Engineering, and Medicine reported conclusive or substantial evidence that cannabis or cannabinoids are effective for the treatment of pain in adults; chemotherapy-induced nausea, vomiting, and spasticity associated with multiple sclerosis, and moderate evidence was found for secondary sleep disturbances [29,30].

Most often, attention is focused on the indications of cannabinoid therapy for pain, inflammation, immune disorders, obesity, other metabolic and neurodegenerative changes, and cardiovascular and gastrointestinal diseases, which are often diagnosed in patients with neurodegeneration and dementia-related diseases such as Alzheimer’s, Parkinson’s disease (PD), and Huntington’s [30,31,32].

Of note is a report from the 40th meeting of the World Health Organization’s Expert Committee on Drug Dependence, which stated: “No reports of controlled studies of the physical dependence potential of CBD in laboratory animals or humans could be identified. Tolerance to the effects of CBD has not been observed” [33]. This report is based on the results of controlled preclinical animal studies as well as clinical studies in humans showing no signs of dependence when using CBD. Therefore, when assessing the therapeutic use of cannabinoids, attention is primarily focused on CBD, which should not be associated with any public health problems associated with its therapeutic utilization.

In the treatment of health problems in the elderly, therapy with cannabinoids [21,34,35] or indirect modulators of endocannabinoid mechanisms [26,27] is increasingly recommended for many indications.

It is repeatedly strongly recommended to continue paying attention to the effects of substances that can positively interfere with the mechanisms of endocannabinoids and their functionally related signaling pathways with regulatory therapeutic potential [28]. These therapies are repeatedly evaluated as effective and safe [28,36,37,38,39,40]. Preference is rightly given to the application of CBD, which has been proven not to cause dependence with withdrawal symptoms after the end of treatment [41].

## 3. Pharmacokinetics and Pharmacodynamics of Cannabinoids

Phytocannabinoid CBD is a highly lipophilic substance with a large volume of distribution (Vd~32 L/kg) and poor bioavailability after oral administration (estimated to be as low as 6%). It undergoes, given orally, a significant hepatic first-pass effect in terms of pre-systemic elimination, and a large part of the dose is excreted unchanged in the feces [42,43,44,45]. It has been shown that the AUC and Cmax were CBD dose-dependent, and Tmax mostly occurred between 1 and 4 h [46]. Cannabidiol quickly distributes into well-vascularized organs such as the lung, heart, brain, and liver. It is quickly redistributed to the brain and fat tissue [45,47,48]. CBD may accumulate in adipose tissues following chronic administration. It is metabolized, particularly in the liver, by isozymes CYP2C19 and CYP3A4 and additionally by CYP1A1, CYP1A2, CYP2C9, and CYP2D6. The elimination half-life ranged from 2 to 5 days after chronic oral administration [45,49]; it was reported between 1.4 and 10.9 h after oromucosal spray, 24 h after i.v., and 31 h after smoking (single doses) [46].

The pharmacodynamics of CBD are based particularly on receptor-mediated mechanisms of action with a whole variety of modulated receptors; nevertheless, it has also been shown recently that some indirect mechanisms are involved. The principal modes consist of negative allosteric modulation of cannabinoid CB1 receptors (leading to decreased efficacy of agonists acting on these receptors, such as endogenous cannabinoid anandamide or phytocannabinoid delta-9-tetrahydrocannabinol) and antagonism/inverse agonism on cannabinoid CB2 receptors [50,51]. Moreover, antagonism at the receptor GPR55 has been described. It has been shown that, concerning the aforementioned structures, CBD also acts as an inverse agonist/antagonist on cannabinoid CB1 receptors and a negative allosteric modulator on cannabinoid CB2 receptors [51].

**Figure 1:**
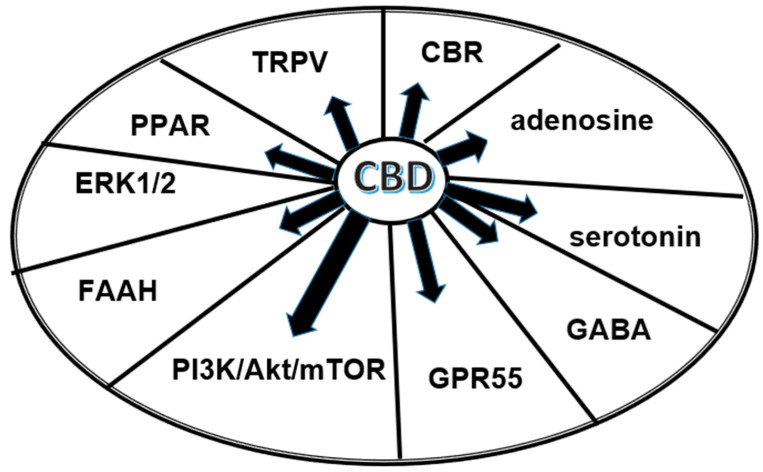
Major substances and structures influenced by CBD.

Besides effects on well-established cannabinoid receptors, CBD also affects many receptors in other receptor systems (Figure 1). Hence, it has been described that CBD acts as an agonist on serotonergic receptor 5-HT1A, a partial agonist on subtype 5-HT2A, and a non-competitive antagonist on subtype 5-HT3A. It activates the adenosine receptors A1, GABAergic GABAA receptors, and the nuclear receptor PPARγ. CBD is an inverse agonist for the G-protein-coupled receptors 3, 6, and 12 (GPR3, GPR6, and GPR12) [21,52]. Furthermore, it stimulates α1 and α1β subunits of the glycine receptors and has a partially agonistic effect on dopamine D2 receptors. It has agonistic activity on the ionotropic receptors TPPA1, TRPV1, TRPV2, TRPV3, TRPV4, and TRPV8 [21,51]. It has been found that CBD also binds to the μ-opioid receptor (MOR) and the δ-opioid receptor (DOR) [53]. There is also developing discussion about possible non-receptor mechanisms such as an increase in anandamide levels (which is thought to be in context with the CBD inhibitory effect on the inactivating hydrolase FAAH) [51,54,55,56,57,58] or non-selective inhibition of the voltage-dependent potassium channels [57].

A network-based pharmacological analysis was used to predict the potential molecular targets for CBD’s anti-inflammatory effects, which revealed the NFκB cascade as one of its primary anti-inflammatory mechanisms of action. Moreover, target proteins, including p53, IκBα, IKKs, and MAP kinases, as well as signaling pathways, including STAT3, AKT1, TNF, TLR, RLRs, and MAPK, were linked to CBD’s anti-inflammatory influence. These molecular targets could contribute to CBD’s overall anti-inflammatory activity and its potential therapeutic significance for various inflammatory-mediated diseases. Although further biological experiments are needed to confirm these molecular targets, these results speak in favor of data supporting the utilization of CBD as a promising anti-inflammatory natural substance [59].

## 4. Examples of Therapeutical Effects of CBD in Clinical Practice

As described above, CBD affects a large number of receptor types and subtypes, so it is not surprising that its documented actions are very different and were described on both preclinical and clinical levels. Initiatory clinical evidence suggests that CBD has a suitable safety profile [60], and the three best-identified effects of CBD are summarized in the following paragraphs.

Probably the most frequently mentioned and widely accepted indication for CBD is its anti-epileptic effect, and it is considered an effective anti-seizure medication. Its exact mechanism of action in these indications is not fully understood. However, CBD is known to reduce neuronal hyperexcitability by modulating intracellular calcium via G protein-coupled receptor 55 (GPR55) and transient receptor potential vanilloid 1 (TRPV-1); adenosine-mediated signaling modulation by inhibition of cellular adenosine absorption via nucleoside transporters-1 (ENT-1) has also been reported.

The effects of CBD were observed on spasm attacks within Dravet syndrome (a serious epileptic disorder typically seen in childhood associated with seizures resistant to therapy and a high mortality rate) in a double-blind, placebo-controlled study carried out with 120 patients. CBD was administered as a supplement to the standard epileptic treatment and decreased the frequency of the spasms in both the children and adolescents suffering from Dravet syndrome for 14 days [61].

Already, a “legendary” case report described the administration of CBD to a 5-year-old girl. This child experienced the first seizure in the form of a prolonged status epilepticus at 3 months of age, and therapy with “classical” anti-epileptic drugs completely failed. The girl was given cannabis extract with a high content of CBD, which led to a reduced seizure frequency from nearly 50 convulsive attacks per day to 2–3 convulsions during nights per month (i.e., >90% reduction in generalized tonic–clonic seizures) [62].

Finally, CBD was approved by the Food and Drug Administration (FDA) in 2018 as an add-on antiepileptic drug in ≥2-year-old children with Dravet syndrome and Lennox–Gastaut syndrome (symptomatic epilepsy caused by brain injury or malformation with manifestations up to 5 years of age), and it was also approved by the European Medicines Agency (EMA) in 2019 [63]. A registered, ready-made preparation with the brand name Epidyolex in the form of an oral solution is now available.

Other reports describing the effects of CBD on epilepsy can be found in the following papers [63,64,65].

The second domain of potential indications for CBD use under discussion is chronic pain. Nevertheless, according to our knowledge and the following literary sources, no approved pharmaceutical products containing CBD alone for the management of pain are available. For pain management, cannabinoids are usually administered as medical hemp or nabiximols (which are a combined product of THC/CBD in a 1:1 ratio), and it may therefore be difficult to unambiguously attribute the therapeutic properties of CBD alone since it is administered with THC [66]. Moreover, many CBD products are presently available as supplements, but these products are non-pharmaceuticals, and there is a lack of appropriate clinical studies supporting their efficacy [66].

Despite this limitation, the first reports available suggest the possible benefit of CBD use for pain treatment. Eskander et al. described the positive effects of CBD in the form of cream on acute and chronic back pain on the basis of case reports. They concluded that modulation of the noradrenergic system and indirect stimulation of opioid receptors via CB2 receptors can play an important role in pain management [67].

Xu et al. focused on the possible benefit of CBD treatment in patients with symptomatic peripheral neuropathy. The study involved 29 patients. Fifteen patients were administered CBD (250 mg/3 fl. oz) and 14 patients were given a placebo. After four weeks, the placebo group was allowed to crossover into the treatment group. For the pain assessment, the neuropathic pain scale (NPS) was administered biweekly. It has been found that a statistically significant reduction in intense pain, sharp pain, and cold and itchy sensations was seen in the CBD group compared to the placebo group. There were no adverse effects reported in this study. The authors concluded that the transdermal application of CBD oil can achieve significant improvement in pain and other disturbing sensations in patients suffering from peripheral neuropathy and that the treatment product was well tolerated [68].

On the other hand, Schneider et al. [69] focused in their randomized, placebo-controlled, double-blinded, crossover study on the assessment of pain intensities (using a numeric rating scale), secondary hyperalgesia (von Frey filament), and allodynia (dry cotton swab) in an acute pain model using intradermal electrical stimulation. This study involved 20 healthy volunteers and compared the effect of 800 mg of orally administered CBD on pain with a placebo. Its results failed to show a significant effect, which indicates a little bit of controversy concerning findings concerning CBD use in pain treatment.

The issue of the possible therapeutic use of CBD in the treatment of chronic pain conditions was recently the subject of a meta-analysis involving a total of 12 studies. CBD is mentioned by the authors as a suitable alternative to opioids because of its good tolerability. From the point of view of efficacy, there is also an emphasis on the beneficial effect on improving sleep and the quality of life of patients. At the same time, however, the authors stress the need for further clinical studies to verify the therapeutic benefit of CBD in this particular indication [70]. Other reports describing the effects of CBD on pain can be found in the following papers [29,71,72].

Finally, the use of CBD is frequently mentioned in association with its possible beneficial effects on anxiety. The effects of CBD were tested in an adolescent with multiple substance use disorders (cannabis, MDMA, cocaine), severe depression, social anxiety, and narcissistic personality disorder. This patient was given capsules in different dosages (starting at 100 mg up to 600 mg over 8 weeks) after unsuccessful treatment with antidepressant medication. Antidepressant medication was ceased, and following the CBD treatment, the patient both improved her depressive and anxiety symptoms [73].

The efficacy of CBD was investigated in association with anxiety disorders in young people who previously failed to respond to standard therapy. The study was open-label and involved 31 young people (12–25 years) with anxiety disorder who showed no clinical improvement after treatment with cognitive-behavioral therapy and/or antidepressant drugs. All patients were given add-on CBD for 12 weeks on a fixed-flexible schedule titrated up to 800 mg/d. Anxiety was assessed with the overall anxiety severity and impairment scale (OASIS) at week 12. Mean (SD) OASIS scores decreased from 10.8 (3.8) at baseline to 6.3 (4.5) at week 12, corresponding to a −42.6% reduction (*p*  <  0.0001). These results suggest that CBD reduces anxiety severity in young people suffering from treatment-resistant anxiety disorders [74].

A systematic review concerning human studies assessed the efficacy of cannabidiol for social anxiety. The authors of this very recent paper concluded that CBD may be a promising treatment for this condition, despite the fact that further research is needed to establish optimal dosing and assess the time course of CBD’s anxiolytic effects [75]. Other reports describing the effects of CBD on anxiety can be found in the following papers [72,73,76,77,78].

**Figure 2:**
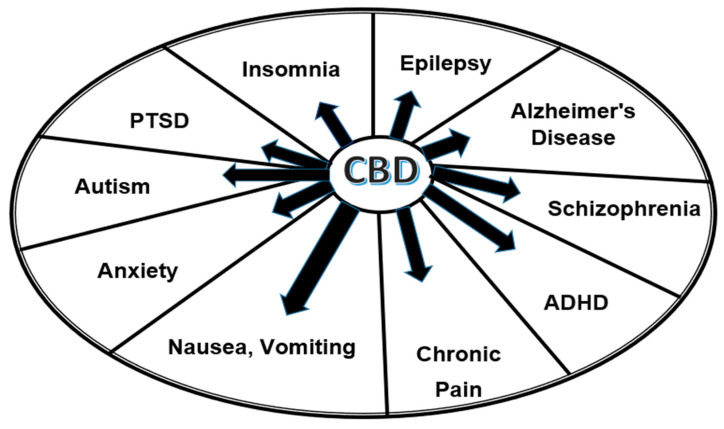
The most promising treatment indications of CBD.

Besides these three indications, available medical reports gradually confirm the potential therapeutic effects of CBD for many other purposes, including anorexia [29], cancer [71], ulcerative colitis [79], dementia [80], depression [72,73,81], dyslipidemia [78], schizophrenia [78,82], sleep disorders [29,72], substance abuse [73], spasticity in multiple sclerosis [29], autism and associated disorders [83,84], attention deficit/hyperactivity disorder (ADHD), posttraumatic stress disorder (PTSD) [85], etc. The most promising indications are shown in Figure 2.

The published results of an increasing number of not only preclinical but also clinical research studies repeatedly confirm the therapeutic potential of CBD in various pathological conditions. Therefore, it is recommended to continue “de lege artis medicinae” clinical studies with the application of CBD in various indications. The positive results could be subsequently applied to regulatory guidelines for the therapeutic use of CBD in clinical human medicine [86].

## 5. Effects of CBD on Aging Including Alzheimer’s Disease

Aging is a universal process of molecular and physiological changes that are significantly associated with susceptibility to disease and eventually lead to death [87]. Due to the involvement of the endocannabinoid system (ECS) in many physiological and regulatory functions and roles, there are many reports on the influence of the ECS on this process. For example, it has been described that enhancing the endogenous cannabinoid tone may induce beneficial effects on the evolution of Alzheimer’s disease [30] or that ECS regulates numerous aspects of circadian physiology relevant to the neurobiology of aging [22]. The progression of aging is determined by the balance between damaging processes contributing to aging and the activity of the homeostatic defense mechanisms, and ECS is believed to be part of these latter actions [19]. With an aging population globally, there is an increase in age-related diseases, such as Alzheimer’s disease, resulting in cognitive decline. Therefore, a focus on more efficient and disease-modifying therapeutic approaches that could delay or mitigate disease progression is necessary [32], and since the pharmacological profile and effects of CBD within ECS are similar, it can be expected that this substance could significantly influence the process of aging.

Disruption in protein homeostasis plays, together with oxidative stress and neuroinflammation, a significant role in the pathobiology of dementia disorders. Cannabidiol is known for its ability to decrease oxidative stress, neuroinflammation, and protein misfolding, and it plays a role in various preclinical and clinical models of neurological disorders, which suggests its potential therapeutic use in this field [88].

It has been shown in pre-clinical studies that CBD improves both spatial and recognition memory and decreases anxiety in a mouse model of Alzheimer’s disease. Mice (SAMP8) develop an age-related impairment in learning and memory corresponding to an increase in amyloid-β, hyperphosphorylated tau, oxidative stress, impaired efflux of amyloid-β across the blood–brain barrier, and neuroinflammation. These mice, starting at 11 months of age, were administered CBD (0, 3, or 30 mg/kg) daily orally for 60 days. After thirty days of treatment, the following parameters were tested: learning and memory (T-maze and novel object recognition), activity in an open field, and anxiety in the elevated plus maze. Mice that were given CBD at 30 mg/kg significantly improved learning and memory in the T-maze compared to the control group, and CBD also led to improved novel object recognition memory. The effect was probably due to the antioxidant properties of CBD, which suggest cannabidiol as a potential treatment for age-related dementia [89]. Furthermore, similar results were obtained in another study in 14-month-old TAU58/2 transgenic mice, in which chronically administered CBD significantly improved impaired spatial reference memory, suppressed anxiety-like behavior, and reduced contextual fear-associated freezing. Therefore, the authors of the study suggested the potential of its use in the treatment of tauopathy-related behavioural impairments including cognitive deficits [90].

A very recent study investigated the memory-enhancing, anti-inflammatory, and anti-aging potential of CBD in vitamin D3-deficient diet-induced rats. It was found that CBD interacted with CYP2R1, CYP27B1, CYP24A1, and vitamin D receptors. This led to increased vitamin D3 metabolites, which improved memory and reduced inflammation and aging through modulating antioxidative enzymes (superoxide dismutase (SOD) and glutathione peroxidase (GPx)), cytokines, and neurotransmitters in these rats [91,92].

A very interesting insight into the possible benefit of CBD in pathologies characterized by memory impairment was provided by a recent preclinical proof-of-concept study looking at the effect of CBD on brain metabolism in a streptozotocin-induced Alzheimer’s disease rat model. Compared to the control group of animals (saline), positron emission tomography showed a significantly higher level of glucose metabolism in the lateral ventricle area. Significant differences in metabolic levels were additionally observed, mainly in the striatum, motor cortex, hippocampus, and thalamus. The observed effects could be particularly important when considering that lower glucose utilization is one of the early and persistent signs of Alzheimer’s dementia [93].

Cannabinoids, through CB2 receptors, affect microglia activity in an age-dependent manner [23]. It has been shown that the high-affinity agonists of TRPV2, including CBD, significantly increase the ability of microglia to phagocytose amyloid-beta by activating TRPV2; blockade of these receptors has the opposite effect. In addition to suppressing neuroinflammation, CBD also significantly improves mitochondrial function and adenosine triphosphate (ATP) production, again in correlation with TRPV2 activity [94,95].

Interest was also given to the potentiality of CBD acting as a pharmacological modulator of the protein homeostasis network, particularly in terms of its neuroprotective and aggregate clearing roles in neurodegenerative disorders. It has been concluded that CBD could be a useful modulator for reversing not only age-associated neurodegeneration (proteinopathies, including Alzheimer’s disease, Parkinson’s disease, and Huntington’s disease) but also other protein misfolding disorders. The present understanding of the role of CBD is nevertheless insufficient to support this suggestion unambiguously [88]. Similarly, another review focused on the modulatory effects of CBD on Alzheimer’s disease gives a summary that for intervening in Alzheimer’s disease pathology and for the translation of preclinical studies into clinical settings, understanding the underlying mechanisms of CBD is necessary [96].

Currently, we also have clinical data on the use of CBD in the treatment of patients with already developed dementia, although these are still smaller groups of patients. An example is a randomized, double-blind, placebo-controlled study involving 60 patients diagnosed with major neurocognitive disorders and associated behavioral disturbances. They were randomized to receive a placebo of cannabis oil Avidekel (295 mg CBD and 12.5 mg THC per milliliter) three times daily for 16 weeks. The primary assessed study endpoint was a decrease in the Cohen–Mansfield Agitation Inventory score. A decrease of at least four points at the end of the study was achieved in 60% of patients treated with cannabis oil, but only in 30% in the placebo arm (*p* = 0.03). There was also a significant difference in the number of patients achieving a decrease of at least eight points, i.e., 50% versus 15% of patients (*p* = 0.01). Treatment was very well tolerated by patients with similar adverse event profiles in both arms of the study [97]. Also, another smaller study (*n* = 20) mentions a therapeutic benefit of 3% CBD in the treatment of severe behavioral and psychological symptoms of dementia [98], and further studies are planned [99].

Previously, the Caenorhabditis elegans model showed that life expectancy is closely related to the activity of genes responsible for autophagy, a lysosomal catabolic process that determines cellular homeostasis and is also related to neuroprotection at the level of the central nervous system [100,101]. Recently published papers describe quite convincingly the ability of CBD to regulate the process of autophagy, although the exact mechanism of this regulation has not yet been fully understood. For example, at the level of cell lines of human neuroblastoma SH-SY5Y or mouse astrocytes, CBD-induced autophagy has been shown to be significantly lower in the presence of CB1, CB2, and TRPV1 (Transient Receptor Potential Vanilloid 1) antagonists. CBD-induced autophagy in this model was mediated by activation of ERK1/2 (extracellular signal-regulated protein kinases 1 and 2) and suppression of protein kinase B (AKT), key regulators of cell proliferation and survival. The induction of autophagy itself was dependent on Unc51-like kinase (ULK1) [102].

However, the process of autophagy is also significantly regulated by the SIRT1 gene, which encodes sirtuin 1 [103]. Recent experimental work with C. elegans confirms CBD-induced autophagy in hippocampal cells and SH-SY5Y neurons while extending lifespan in direct dependence on the autophagic genes bec-1, vps-34, and sqst-1 [104]. In addition, CBD also suppresses age-related changes in the morphology (beading and blebbing) of the touch receptor neurons (TRNs). These effects are linked to the aforementioned SIRT1 [104].

Furthermore, CBD added to the cell culture of SH-SY5Y neurons prevented damage to neurites by amyloid Aβ1-42 and led to higher expression of fatty acid amide hydrolase (FAAH) and the CB1 receptor. At the same time, it acted protectively in terms of limiting the decreasing density of dendrites [105].

## 6. Adverse Effects of CBD and Limitations of Its Use

One of the most frequently seen adverse effects of CBD is dose-dependent hepatotoxicity. For example, it has been reported that serum aminotransferase elevations arose during CBD therapy for epilepsy in 34% to 47% of patients, compared to 18% of controls who were given other anticonvulsant drugs. The cause of this elevation was not known; nevertheless, it could occur due to direct toxicity (either the molecule itself or the production of a toxic intermediate in its metabolism) [106]. Another study investigating the safety and tolerability of CBD and its effect on common parkinsonian symptoms also showed that the relatively high dose used in this study was associated with liver enzyme elevations [107]. Finally, a very recent meta-analysis aimed to determine the association between cannabidiol use, liver enzyme elevation, and drug-induced liver injury concluded that CBD-associated liver enzyme elevations and drug-induced liver injury met the criteria of common adverse drug events, and therefore monitoring of liver function in patients at increased risk was recommended during CBD use [108].

Despite the many beneficial effects of CBD (particularly on conditions occurring in the elderly) and its possible therapeutic indications, it is without doubt that CBD use is associated with some limitations. For example, a retrospective observational study suggested that CBD-rich treatment has a beneficial impact on pain, anxiety, and depression symptoms as well as overall wellbeing only for patients with moderate to severe symptoms; however, there was no observed effect on mild symptoms [109]. Moreover, quite frequently, clinical effects were seen and tested only in small clinical trials, and further investigation is therefore demanded.

## 7. Conclusions

Despite the aforementioned limitations, there is an increasing number of not only preclinical but also clinical research studies (yet small) confirming repeatedly the therapeutic potential of CBD in various pathological conditions. It is recommended to continue with “lege artis” clinical studies, providing the results that could be utilized in regulatory guidelines for therapeutic applications of CBD in clinical medical care in humans [86].

According to all the results of the available clinical studies, CBD has shown clear therapeutic efficacy in the elderly human population. The frequency of side effects is comparable to other classes of drugs, and the risks of using CBD in elderly patients are rated as moderate. However, in geriatric patients, the presence of (a) increased cardiovascular risk, (b) pharmacokinetic changes, and (c) possible interactions with existing, often polypharmaceutical therapy, must be considered individually [36,37].

## Data Availability

Not applicable.

## References

[1] Kirkwood T.B.L. (2005). Understanding the Odd Science of Aging. Cell.

[2] Di Marzo V., Wang J. (2014). The Endocannabinoidome: The World of Endocannabinoids and Related Mediators.

[3] Piomelli D. (2003). The molecular logic of endocannabinoid signalling. Nat. Rev. Neurosci..

[4] Di Marzo V., Piscitelli F. (2015). The Endocannabinoid System and its Modulation by Phytocannabinoids. Neurotherapeutics.

[5] Pertwee R.G. (2005). Pharmacological actions of cannabinoids. Handbook of Experimental Pharmacology.

[6] Paradisi A., Oddi S., Maccarrone M. (2006). The Endocannabinoid System in Ageing: A New Target for Drug Development. Curr. Drug Targets.

[7] Micale V., Di Marzo V., Sulcova A., Wotjak C.T., Drago F. (2013). Endocannabinoid system and mood disorders: Priming a target for new therapies. Pharmacol. Ther..

[8] Beedham W., Sbai M., Allison I., Coary R., Shipway D. (2020). Cannabinoids in the Older Person: A Literature Review. Geriatrics.

[9] Bryk M., Starowicz K. (2021). Cannabinoid-based therapy as a future for joint degeneration. Focus on the role of CB2 receptor in the arthritis progression and pain: An updated review. Pharmacol. Rep..

[10] Mangieri R.A., Piomelli D. (2007). Enhancement of endocannabinoid signaling and the pharmacotherapy of depression. Pharmacol. Res..

[11] Paredes-Ruiz K.J., Chavira-Ramos K., Orozco-Morales M., Karasu C., Tinkov A.A., Aschner M., Santamaría A., Colín-González A.L. (2021). On the Biomedical Properties of Endocannabinoid Degradation and Reuptake Inhibitors: Pre-clinical and Clinical Evidence. Neurotox. Res..

[12] Landa L., Jurica J., Sliva J., Pechackova M., Demlova R. (2018). Medical cannabis in the treatment of cancer pain and spastic conditions and options of drug delivery in clinical practice. Biomed. Pap..

[13] Trojan V., Landa L., Hrib R., Jurica J., Rychlickova J., Zvonicek V., Halamkova L., Halamek J., Demlova R., Belaskova S. (2022). Assessment of Delta-9-Tetrahydrocannabinol (THC) in Saliva and Blood After Oral Administration of Medical Cannabis With Respect to its Effect on Driving Abilities. Physiol. Res..

[14] Fisar Z. (2009). Phytocannabinoids and endocannabinoids. Curr. Drug Abus. Rev..

[15] Zendulka O., Dovrtělová G., Nosková K., Turjap M., Šulcová A., Hanuš L., Juřica J. (2016). Cannabinoids and Cytochrome P450 Interactions. Curr. Drug Metab..

[16] Mackie K. (2006). Cannabinoid Receptors as Therapeutic Targets. Annu. Rev. Pharmacol. Toxicol..

[17] Izzo A.A., Borrelli F., Capasso R., Di Marzo V., Mechoulam R. (2009). Non-psychotropic plant cannabinoids: New therapeutic opportunities from an ancient herb. Trends Pharmacol. Sci..

[18] Battista N., Di Tommaso M., Bari M., Maccarrone M. (2012). The endocannabinoid system: An overview. Front. Behav. Neurosci..

[19] Bilkei-Gorzo A. (2012). The endocannabinoid system in normal and pathological brain ageing. Philos. Trans. R. Soc. B Biol. Sci..

[20] Whiting P.F., Wolff R.F., Deshpande S., Di Nisio M., Duffy S., Hernandez A.V., Keurentjes J.C., Lang S., Misso K., Ryder S. (2015). Cannabinoids for Medical Use: A Systematic Review and Meta-analysis. JAMA.

[21] Sulcova A. (2019). Pharmacodynamics of cannabinoids. Arch. Pharm. Pharm. Sci..

[22] Hodges E.L., Ashpole N.M. (2019). Aging circadian rhythms and cannabinoids. Neurobiol. Aging.

[23] Komorowska-Müller J.A., Rana T., Olabiyi B.F., Zimmer A., Schmöle A.-C. (2021). Cannabinoid Receptor 2 Alters Social Memory and Microglial Activity in an Age-Dependent Manner. Molecules.

[24] Martinelli G., Magnavacca A., Fumagalli M., Dell’agli M., Piazza S., Sangiovanni E. (2021). *Cannabis sativa* and Skin Health: Dissecting the Role of Phytocannabinoids. Planta Medica.

[25] Ortega-Gutierrez S. (2005). Therapeutic perspectives of inhibitors of endocannabinoid degradation. Curr. Drug Target CNS Neurol. Disord..

[26] de Lago E., Fernández-Ruiz J., Ortega-Gutiérrez S., Cabranes A., Pryce G., Baker D., López-Rodríguez M., Ramos J.A. (2006). UCM707, an inhibitor of the anandamide uptake, behaves as a symptom control agent in models of Huntington’s disease and multiple sclerosis, but fails to delay/arrest the progression of different motor-related disorders. Eur. Neuropsychopharmacol..

[27] de Lago E., Ortega-Gutiérrez S., Ramos J.A., Rodríguez M.L.L., Fernández-Ruiz J. (2007). Neurochemical effects of the endocannabinoid uptake inhibitor UCM707 in various rat brain regions. Life Sci..

[28] Piomelli D., Tagne A.M. (2022). Endocannabinoid-Based Therapies. Annu. Rev. Pharmacol. Toxicol..

[29] Abrams D.I. (2018). The therapeutic effects of Cannabis and cannabinoids: An update from the National Academies of Sciences, Engineering and Medicine report. Eur. J. Intern. Med..

[30] Benito C., Núñez E., Pazos M.R., Tolón R.M., Romero J. (2007). The Endocannabinoid System and Alzheimer’s Disease. Mol. Neurobiol..

[31] Maccarrone M., Gasperi V., Catani M.V., Diep T.A., Dainese E., Hansen H.S., Avigliano L. (2010). The Endocannabinoid System and Its Relevance for Nutrition. Annu. Rev. Nutr..

[32] Dos Reis Rosa Franco G., Smid S., Viegas C. (2021). Phytocannabinoids: General Aspects and Pharmacological Potential in Neurodegenerative Diseases. Curr. Neuropharmacol..

[33] https://apps.who.int/iris/bitstream/handle/10665/279948/9789241210225-eng.pdf.

[34] Crippa J.A., Guimarães F.S., Campos A.C., Zuardi A.W. (2018). Translational Investigation of the Therapeutic Potential of Cannabidiol (CBD): Toward a New Age. Front. Immunol..

[35] Batalla A., Janssen H., Gangadin S.S., Bossong M.G. (2019). The Potential of Cannabidiol as a Treatment for Psychosis and Addiction: Who Benefits Most? A Systematic Review. J. Clin. Med..

[36] Abuhasira R., Schleider L.B.-L., Mechoulam R., Novack V. (2018). Epidemiological characteristics, safety and efficacy of medical cannabis in the elderly. Eur. J. Intern. Med..

[37] Abuhasira R., Ron A., Sikorin I., Novack V. (2019). Medical Cannabis for Older Patients—Treatment Protocol and Initial Results. J. Clin. Med..

[38] Solomon H.V., Greenstein A.P., DeLisi L.E. (2021). Cannabis Use in Older Adults: A Perspective. Harv. Rev. Psychiatry.

[39] Morel A., Lebard P., Dereux A., Azuar J., Questel F., Bellivier F., Marie-Claire C., Fatséas M., Vorspan F., Bloch V. (2021). Clinical Trials of Cannabidiol for Substance Use Disorders: Outcome Measures, Surrogate Endpoints, and Biomarkers. Front. Psychiatry.

[40] Sulcova A. (2021). Translational research on the effects of treatment with cannabidiol in addictions. Adiktologie.

[41] Taylor L., Crockett J., Tayo B., Checketts D., Sommerville K. (2020). Abrupt withdrawal of cannabidiol (CBD): A randomized trial. Epilepsy Behav..

[42] Wall M.E., Brine D.R., Perez-Reyes M., Braude M.C., Szara S. (1976). The Pharmacology of Marihuana.

[43] Agurell S., Carlsson S., Lindgren J.E., Ohlsson A., Gillespie H., Hollister L. (1981). Interactions of delta 1-tetrahydrocannabinol with cannabinol and cannabidiol following oral administration in man: Assay of cannabinol and cannabidiol by mass fragmentography. Experientia.

[44] Huestis M.A. (2007). Human Cannabinoid Pharmacokinetics. Chem. Biodivers.

[45] Lucas C.J., Galettis P., Schneider J. (2018). The pharmacokinetics and the pharmacodynamics of cannabinoids. Br. J. Clin. Pharmacol..

[46] Millar S.A., Stone N.L., Yates A.S., O’Sullivan S.E. (2018). A Systematic Review on the Pharmacokinetics of Cannabidiol in Humans. Front. Pharmacol..

[47] Ohlsson A., Lindgren J.-E., Andersson S., Agurell S., Gillespie H., Hollister L.E. (1986). Single-dose kinetics of deuterium-labelled cannabidiol in man after smoking and intravenous administration. J. Mass Spectrom..

[48] Gaston T.E., Friedman D. (2017). Pharmacology of cannabinoids in the treatment of epilepsy. Epilepsy Behav..

[49] Consroe P., Laguna J., Allender J., Snider S., Stern L., Sandyk R., Kennedy K., Schram K. (1991). Controlled clinical trial of cannabidiol in Huntington’s disease. Pharmacol. Biochem. Behav..

[50] Mielnik C.A., Lam V.M., Ross R.A. (2021). CB1 allosteric modulators and their therapeutic potential in CNS disorders. Prog. Neuro-Psychopharmacol. Biol. Psychiatry.

[51] Peng J., Fan M., An C., Ni F., Huang W., Luo J. (2022). A narrative review of molecular mechanism and therapeutic effect of cannabidiol (CBD). Basic Clin. Pharmacol. Toxicol..

[52] Laun A.S., Shrader S.H., Brown K.J., Song Z.H. (2019). GPR3. GPR6, and GPR12 as novel molecular targets: Their biological functions and interaction with cannabidiol. Acta Pharmacol. Sin..

[53] Mlost J., Bryk M., Starowicz K. (2020). Cannabidiol for Pain Treatment: Focus on Pharmacology and Mechanism of Action. Int. J. Mol. Sci..

[54] Laprairie R.B., Bagher A.M., Kelly M.E., Denovan-Wright E.M. (2015). Cannabidiol is a negative allosteric modulator of the cannabinoid CB1 receptor. Br. J. Pharmacol..

[55] Morales P., Hurst D.P., Reggio P.H. (2017). Molecular targets of the phytocannabinoids: A complex picture. Prog. Chem. Org. Nat. Prod..

[56] Preedy V.R. (2017). Handbook of Cannabis and Related Pathologies: Biology, Pharmacology, Diagnosis, and Treatment.

[57] Ghovanloo M.R., Shuart N.G., Mezeyova J., Dean R.A., Ruben P.C., Goodchild S.J. (2018). Inhibitory effects of cannabidiol on voltage-dependent sodium currents. J. Biol. Chem..

[58] Rodríguez-Muñoz M., Onetti Y., Cortés-Montero E., Garzón J., Sánchez-Blázquez P. (2018). Cannabidiol enhances morphine antinociception, diminishes NMDA-mediated seizures and reduces stroke damage via the sigma 1 receptor. Mol. Brain.

[59] Ma H., Xu F., Liu C., Seeram N.P. (2021). A Network Pharmacology Approach to Identify Potential Molecular Targets for Cannabidiol’s Anti-Inflammatory Activity. Cannabis Cannabinoid Res..

[60] Iffland K., Grotenhermen F., Reddon H., DeBeck K., Socias M.-E., Lake S., Dong H., Hayashi K., Milloy M.-J., Wright M. (2017). An Update on Safety and Side Effects of Cannabidiol: A Review of Clinical Data and Relevant Animal Studies. Cannabis Cannabinoid Res..

[61] Devinsky O., Cross J.H., Laux L., Marsh E., Miller I., Nabbout R., Scheffer I.E., Thiele E.A., Wright S., Cannabidiol in Dravet Syndrome Study Group (2017). Trial of cannabidiol for drug-resistant seizures in the Dravet syndrome. N. Engl. J. Med..

[62] Maa E., Figi P. (2014). The case for medical marijuana in epilepsy. Epilepsia.

[63] Raucci U., Pietrafusa N., Paolino M.C., Di Nardo G., Villa M.P., Pavone P., Terrin G., Specchio N., Striano P., Parisi P. (2020). Cannabidiol Treatment for Refractory Epilepsies in Pediatrics. Front. Pharmacol..

[64] Fong S.L., Kossoff E.H. (2018). Cannabinoids as future treatment for epilepsy. Contemp. PEDS J..

[65] Pietrafusa N., Ferretti A., Trivisano M., de Palma L., Calabrese C., Pavia G.C., Tondo I., Cappelletti S., Vigevano F., Specchio N. (2019). Purified Cannabidiol for Treatment of Refractory Epilepsies in Pediatric Patients with Developmental and Epileptic Encephalopathy. Pediatr. Drugs.

[66] Boyaji S., Merkow J., Elman R.N.M., Kaye A.D., Yong R.J., Urman R.D. (2020). The Role of Cannabidiol (CBD) in Chronic Pain Management: An Assessment of Current Evidence. Curr. Pain Headache Rep..

[67] Eskander J.P., Spall J., Spall A., Shah R.V., Kaye A.D. (2020). Cannabidiol (CBD) as a treatment of acute and chronic back pain: A case series and literature review. J. Opioid Manag..

[68] Xu D.H., Cullen B.D., Tang M., Fang Y. (2020). The Effectiveness of Topical Cannabidiol Oil in Symptomatic Relief of Peripheral Neuropathy of the Lower Extremities. Curr. Pharm. Biotechnol..

[69] Schneider T.M., Zurbriggen L., Dieterle M., Mauermann E.M., Frei P., Mercer-Chalmers-Bender K., Ruppen W.M. (2022). Pain response to cannabidiol in induced acute nociceptive pain, allodynia, and hyperalgesia by using a model mimicking acute pain in healthy adults in a randomized trial (CANAB I). Pain.

[70] Villanueva M.R.B., Joshaghani N., Villa N., Badla O., Goit R., Saddik S.E., Dawood S.N., Rabih A.M., Niaj A., Raman A. (2022). Efficacy, Safety, and Regulation of Cannabidiol on Chronic Pain: A Systematic Review. Cureus.

[71] Blake A., Wan B.A., Malek L., DeAngelis C., Diaz P., Lao N., Chow E., O’hearn S. (2017). A selective review of medical cannabis in cancer pain management. Ann. Palliat. Med..

[72] Corroon J., Phillips J.A. (2018). A Cross-Sectional Study of Cannabidiol Users. Cannabis Cannabinoid Res..

[73] Laczkovics C., Kothgassner O.D., Felnhofer A., Klier C.M. (2020). Cannabidiol treatment in an adolescent with multiple substance abuse, social anxiety and depression. Neuropsychiatrie.

[74] Berger M., Li E., Rice S., Davey C.G., Ratheesh A., Adams S., Jackson H., Hetrick S., Parker A., Spelman T. (2022). Cannabidiol for Treatment-Resistant Anxiety Disorders in Young People: An Open-Label Trial. J. Clin. Psychiatry.

[75] Fliegel D.K., Lichenstein S.D. (2022). Systematic literature review of human studies assessing the efficacy of cannabidiol for social anxiety. Psychiatry Res. Commun..

[76] Zuardi A.W. (2008). Cannabidiol: From an inactive cannabinoid to a drug with wide spectrum of action. Rev. Bras. Psiquiatr..

[77] de Mello Schier A.R., de Oliveira Ribeiro N.P., Coutinho D.S., Machado S., Arias-Carrión O., Crippa J.A., Zuardi A.W., Nardi A.E., Silva A.C. (2014). Antidepressant-like and anxiolytic-like effects of cannabidiol: A chemical compound of Cannabis sativa. CNS Neurol. Disord. Drug Targets..

[78] Larsen C., Shahinas J. (2020). Dosage, Efficacy and Safety of Cannabidiol Administration in Adults: A Systematic Review of Human Trials. J. Clin. Med. Res..

[79] Irving P.M., Iqbal T., Nwokolo C., Subramanian S., Bloom S., Prasad N., Hart A., Murray C., Lindsay J.O., Taylor A. (2018). A Randomized, Double-blind, Placebo-controlled, Parallel-group, Pilot Study of Cannabidiol-rich Botanical Extract in the Symptomatic Treatment of Ulcerative Colitis. Inflamm. Bowel Dis..

[80] Bosnjak Kuharic D., Markovic D., Brkovic T., Jeric Kegalj M., Rubic Z., Vuica Vukasovic A., Jeroncic A., Puljak L. (2021). Cannabinoids for the Treatment of Dementia. Cochrane Database Syst. Rev..

[81] Sales A.J., Fogaça M.V., Sartim A.G., Pereira V.S., Wegener G., Guimarães F.S., Joca S.R.L. (2018). Cannabidiol Induces Rapid and Sustained Antidepressant-Like Effects Through Increased BDNF Signaling and Synaptogenesis in the Prefrontal Cortex. Mol. Neurobiol..

[82] Ahmed S., Roth R.M., Stanciu C.N., Brunette M.F. (2021). The Impact of THC and CBD in Schizophrenia: A Systematic Review. Front. Psychiatry.

[83] Poleg S., Golubchik P., Offen D., Weizman A. (2018). Cannabidiol as a suggested candidate for treatment of autism spectrum disorder. Prog. Neuro-Psychopharmacol. Biol. Psychiatry.

[84] Pedrazzi J.F.C., Ferreira F.R., Silva-Amaral D., Lima D.A., Hallak J.E.C., Zuardi A.W., Del-Bel E.A., Guimarães F.S., Costa K.C.M., Campos A.C. (2022). Cannabidiol for the treatment of autism spectrum disorder: Hope or hype?. Psychopharmacology.

[85] Kirkland A.E., Fadus M.C., Gruber S.A., Gray K.M., Wilens T.E., Squeglia L.M. (2022). A scoping review of the use of cannabidiol in psychiatric disorders. Psychiatry Res..

[86] Khalsa J.H., Bunt G., Blum K., Maggirwar S.B., Galanter M., Potenza M.N. (2022). Review: Cannabinoids as Medicinals. Curr. Addict. Rep..

[87] Ahadi S., Zhou W., Schüssler-Fiorenza Rose S.M., Sailani M.R., Contrepois K., Avina M., Ashland M., Brunet A., Snyder M. (2020). Personal aging markers and ageotypes revealed by deep longitudinal profiling. Nat. Med..

[88] Dash R., Ali C., Jahan I., Munni Y.A., Mitra S., Hannan A., Timalsina B., Oktaviani D.F., Choi H.J., Moon I.S. (2020). Emerging potential of cannabidiol in reversing proteinopathies. Ageing Res. Rev..

[89] Farr S.A., Goodland M.N., Niehoff M.L., Banerjee S., Young B.J. (2021). Cannabidiol (CBD) improves cognition and decreases anxiety in the SAMP8 mouse model of Alzheimer’s. Alzheimer’s Dement..

[90] Kreilaus F., Przybyla M., Ittner L., Karl T. (2022). Cannabidiol (CBD) treatment improves spatial memory in 14-month-old female TAU58/2 transgenic mice. Behav. Brain Res..

[91] Trivedi M.K., Mondal S., Gangwar M., Jana S. (2022). Effects of Cannabidiol Interactions withCYP2R1, CYP27B1, CYP24A1,and Vitamin D3Receptors on Spatial Memory, Pain, Inflammation, and Aging in Vitamin D3Deficiency Diet-Induced Rats. Cannabis Cannabinoid Res..

[92] Jîtcă G., Ősz B.E., Vari C.E., Rusz C.-M., Tero-Vescan A., Pușcaș A. (2023). Cannabidiol: Bridge between Antioxidant Effect, Cellular Protection, and Cognitive and Physical Performance. Antioxidants.

[93] Faria D.d.P., de Souza L.E., Duran F.L.d.S., Buchpiguel C.A., Britto L.R., Crippa J.A.d.S., Filho G.B., Real C.C. (2022). Cannabidiol Treatment Improves Glucose Metabolism and Memory in Streptozotocin-Induced Alzheimer’s Disease Rat Model: A Proof-of-Concept Study. Int. J. Mol. Sci..

[94] Hassan S., Eldeeb K., Millns P.J., Bennett A.J., Alexander S.P.H., Kendall D.A. (2014). Cannabidiol enhances microglial phagocytosis via transient receptor potential (TRP) channel activation. Br. J. Pharmacol..

[95] Yang S., Du Y., Zhao X., Tang Q., Su W., Hu Y., Yu P. (2022). Cannabidiol Enhances Microglial Beta-Amyloid Peptide Phagocytosis and Clearance via Vanilloid Family Type 2 Channel Activation. Int. J. Mol. Sci..

[96] Xiong Y., Lim C.-S. (2021). Understanding the Modulatory Effects of Cannabidiol on Alzheimer’s Disease. Brain Sci..

[97] Hermush V., Ore L., Stern N., Mizrahi N., Fried M., Krivoshey M., Staghon E., Lederman V.E., Schleider L.B.-L. (2022). Effects of rich cannabidiol oil on behavioral disturbances in patients with dementia: A placebo controlled randomized clinical trial. Front. Med..

[98] Alexandri F., Papadopoulou L., Tsolaki A., Papantoniou G., Athanasiadis L., Tsolaki M. (2023). The Effect of Cannabidiol 3% on Neuropsychiatric Symptoms in Dementia—Six-Month Follow-Up. Clin. Gerontol..

[99] Bartschi J.G., Greenwood L.-M., Montgomery A., Dortants L., Weston-Green K., Huang X.-F., Pai N., Potter J., Schira M.M., Croft R. (2022). Cannabidiol as a Treatment for Neurobiological, Behavioral, and Psychological Symptoms in Early-Stage Dementia: A Double-Blind, Placebo-Controlled Clinical Trial Protocol. Cannabis Cannabinoid Res..

[100] Hashimoto Y., Ookuma S., Nishida E. (2009). Lifespan extension by suppression of autophagy genes in *Caenorhabditis elegans*. Genes Cells.

[101] Braeckman B.P., Vanfleteren J.R. (2007). Genetic control of longevity in *C. Elegans.*. Exp. Gerontol..

[102] Vrechi T.A.M., Leão A.H.F.F., Morais I.B.M., Abílio V.C., Zuardi A.W., Hallak J.E.C., Crippa J.A., Bincoletto C., Ureshino R.P., Smaili S.S. (2021). Cannabidiol induces autophagy via ERK1/2 activation in neural cells. Sci. Rep..

[103] Salminen A., Kaarniranta K. (2009). SIRT1: Regulation of longevity via autophagy. Cell. Signal..

[104] Wang Z., Zheng P., Chen X., Xie Y., Weston-Green K., Solowij N., Chew Y.L., Huang X.-F. (2022). Cannabidiol induces autophagy and improves neuronal health associated with SIRT1 mediated longevity. GeroScience.

[105] Wang Z., Zheng P., Xie Y., Chen X., Solowij N., Green K., Chew Y.L., Huang X.-F. (2021). Cannabidiol regulates CB1-pSTAT3 signaling for neurite outgrowth, prolongs lifespan, and improves health span in Caenorhabditis elegans of Aβ pathology models. FASEB J..

[106] (2012). Cannabidiol. LiverTox: Clinical and Research Information on Drug-Induced Liver Injury [Internet].

[107] Leehey M.A., Liu Y., Hart F., Epstein C., Cook M., Sillau S., Klawitter J., Newman H., Sempio C., Forman L. (2020). Safety and Tolerability of Cannabidiol in Parkinson Disease: An Open Label, Dose-Escalation Study. Cannabis Cannabinoid Res..

[108] Lo L.A., Christiansen A., Eadie L., Strickland J.C., Kim D.D., Boivin M., Barr A.M., MacCallum C.A. (2023). Cannabidiol-associated hepatotoxicity: A systematic review and meta-analysis. J. Intern. Med..

[109] Rapin L., Gamaoun R., El Hage C., Arboleda M.F., Prosk E. (2021). Cannabidiol use and effectiveness: Real-world evidence from a Canadian medical cannabis clinic. J. Cannabis Res..

